# Validation of the Reveal^®^ 3-D for Peanut Lateral Flow Test: AOAC *Performance Tested Method*^SM^ 111901

**DOI:** 10.1093/jaoacint/qsz041

**Published:** 2020-07-23

**Authors:** Quynh-Nhi Le, Alexis Vance, Nawal Bakir, Dave Almy, Emily Slenk, Brooke Roman, Nicole Klass, Benjamin Bastin, Robert Donofrio

**Affiliations:** 1 Neogen Corporation, 620 Lesher Place, Lansing, MI 48912; 2 Q Laboratories, 1930 Radcliff Drive, Cincinnati, OH 45204

## Abstract

**Background:**

Reveal^®^ 3-D for Peanut is an immunochromatographic, lateral flow test for qualitative detection of peanut residue in food manufacturing and food preparation settings. The test can detect low ppm levels of peanut in clean-in-place (CIP) rinses and in swabs from environmental surfaces and can serve as a tool in managing allergen risk.

**Objective:**

The objective of the study was to validate the lateral flow method for detection of peanut in CIP rinses, specifically water, peroxyacetic acid/hydrogen peroxide, and quaternary ammonium compound rinses, and in swabs taken from stainless steel and plastic surfaces.

**Methods:**

CIP rinses spiked with low levels of peanut were tested, as were surfaces inoculated with peanut. Specificity and assay interference were assessed in testing of food commodities with and without added peanut. Assay robustness and test kit stability and consistency testing were also performed.

**Results:**

Results demonstrated that the lateral flow test can detect peanut in CIP rinses in the range of 2–4 ppm and in environmental surface swabs in the range of 3–4 µg/100 cm^2^. Results of specificity testing with 29 common food items showed lack of cross-reactivity, and potential assay interference only from walnut. Data from stability trials supports expiration dating for the kit of up to 23 months post-manufacture.

**Conclusions and Highlights:**

The lateral flow test is a sensitive, specific, and rapid method for detection of low levels of peanut residue in CIP rinses and environmental samples and can be an important component in a comprehensive allergen risk management program.

## Introduction

Peanut allergies are one of the most common causes of severe allergic incidents and the most common cause of food-induced anaphylaxis. These reactions can be caused by direct contact to the skin or digestive system, including through ingestion of products cross-contaminated with peanut or through inhalation of dusts or aerosols containing peanuts. When a severe reaction occurs, administration of epinephrine, for example using an EpiPen^®^, and emergency care are required (1).

Reveal^®^ 3-D for Peanut is an immunochromatographic, lateral flow test device for detection of peanut residue in clean-in-place rinses and swabs from environmental surfaces. The test is intended for use in an industrial food manufacturing or preparation context. It can be used to validate the adequacy of cleaning and/or to identify problem areas such as buildup of peanut residue in processing equipment, filler heads, etc. In rinses, the test can detect peanut residue at 5 ppm. From environmental swabs, 5 µg/100 cm^2^ can be detected. The test is not intended for use in a home or restaurant by peanut-allergic individuals.

## Scope of Method

- *Analyte.*—Peanut.
- *Matrixes.*—Clean-in-place (CIP) rinses, including water, peroxyacetic acid/hydrogen peroxide-based sanitizers [up to 1% (v/v) solution], and quaternary ammonium-based sanitizers [up to 25% (v/v) solution]; environmental swabs from stainless steel and plastic surfaces.
- *Summary of validated performance claims*.—Probability of Detection (POD)
  1. *Water.*—1.0 at 2.8 ppm, 0.9 at 1.0 ppm.
  2. *Peroxyacetic acid/hydrogen peroxide sanitizer.*—1.0 at 2.2 ppm, 0.9 at 0.8 ppm.
  3. *Quaternary ammonium sanitizer.*—1.0 at 4.2 ppm, 0.97 at 2.2 ppm.
  4. *Swab from wet stainless steel.*—1.0 at 3.0 µg/cm^2^, 0.77 at 2.0 µg/cm^2^.
  5. *Swab from dry stainless steel.*—1.0 at 4.0 µg/cm^2^, 0.67 at 1.4 µg/cm^2^.
  6. *Swab from wet plastic.*—1.0 at 3.0 µg/cm^2^, 0.33 at 1.2 µg/cm^2^.
  7. *Swab from dry plastic*.—1.0 at 3.0 µg/cm^2^, 0.27 at 1.2 µg/cm^2^.

## Definitions

- *Probability of detection (POD).*—A statistical analysis that measures the proportion of positive analytical outcomes for a qualitative method for a given matrix at a given analyte level or concentration (2, 3).

## Principle of the Method

Reveal 3-D for Peanut is a lateral flow, immunochromatographic test for detection of peanut proteins. A sample is introduced to the test device where peanut protein (antigen) binds with colloidal gold-conjugated anti-peanut antibodies. The resulting complex flows along a membrane where it encounters a test line coated with anti-peanut antibodies. As the complexed gold particles accumulate, a visible red line is formed. An overload line is included to indicate conditions of antigen excess in the test sample. The overload line is an immobilized mixture of peanut proteins. In the absence (or presence of trace amounts) of peanut in a sample, the anti-peanut gold conjugate binds to the immobilized proteins and forms a visible line. When large amounts of peanut are present in a sample, the antibody gold conjugate will bind the peanut proteins and is inhibited from binding to the overload line. The overload line is carefully balanced to indicate highly positive samples that may cause a hook effect (false-negative result at high concentration) at the test line. Finally, additional reagents are included in the test device to generate a control line, indicating that the test is functioning properly.

## Standard Reference Materials

National Institute of Standards and Technology (NIST; Gaithersburg, MD) SRM 2387 (peanut butter; Sigma-Aldrich Corp., St. Louis, MO) was used to prepare peanut spiking solution.

## Materials and Methods

### Test Kit Information

- *Kit name.*—Reveal^®^ 3-D for Peanut.
- Cat. No.—901041L.
- *Ordering Information.*—Neogen Corp. headquarters, 620 Lesher Place, Lansing, MI 48912. Phone: 800-234-5333 (USA/Canada) or 527-372-9200, Fax: 517-372-2006. foodsafety@neogen.com, www.neogen.com.

### Test Kit Components

- *Reveal 3-D for Peanut test devices.*—Ten, in one foil pouch.
- *Type 9 extraction buffer.*—Ten bottles or sachets, 4 mL each.
- *Sample tubes with caps.*—Ten.
- *Swabs.*—Ten, individually packaged, sterile, with break-off tips.
- *Swab wetting solution.*—One bottle, 10 mL.
- *Instruction leaflet*.

### Other Reagents and Equipment

- *Timer*.

### Safety Precautions

Reagents are for laboratory use only. Kit reagents and test samples should be treated as if they contain peanut constituents and allergic individuals should exercise appropriate caution. A Safety Data Sheet is available from Neogen Corp. Consult with your facility safety advisor for further instructions.

### Precautions

- The test is intended for use in environmental testing in an industrial food manufacturing or preparation context only.
- Do not use test components beyond their expiration date.
- Do not open the foil pouch containing the test devices until just before use.
- Store the test kit at 2–8°C. Avoid freezing.
- Equilibrate the kit to room temperature (18–30°C) before use (for 20–30 min).
- Test extracted samples within 3 h of extraction.

### Test Sample Preparation

#### Rinse samples

- Carefully tear/cut or uncap a sachet or tube of Type 9 extraction buffer and add the entire contents to a sample tube.
- Add 0.25 mL of test sample to the tube containing extraction buffer.
- Secure the tube cap and shake for 1 min.

#### Swab samples

- Carefully tear/cut or uncap a sachet or tube of Type 9 extraction buffer and add the entire contents to a sample tube. For dry surfaces: Remove a sterile swab from the packaging and wet with two drops of swab wetting solution. Swab a 10 x 10 cm area using a crosshatch technique and revolving the swab on the surface. Repeat this swabbing procedure using movements at a right angle to those used in the first swabbing. For other sampling sites (e.g., filler heads) swab as appropriate for the particular location.
- For wet surfaces: Remove a sterile swab from the packaging. Swab a 10 × 10 cm area using a crosshatch technique and revolving the swab on the surface. Repeat this swabbing procedure using movements at a right angle to those used in the first swabbing. For other sampling sites (e.g., filler heads) swab as appropriate for the particular location.
- Return the swab to the sample tube containing extraction buffer and carefully break off the moistened end at the pre-scored mark so that it remains in the tube.
- Secure the cap of the sample tube and shake for 1 min.

### Test Procedure

*Note:* It is essential to place the test device flat on a level surface as soon as liquid has entered the test window to stimulate flow through the device. The test devices are pre-striped with a pale green loading dye in positions T (test), O (overload), and C (control). The loading dye assists with quality and manufacturing checks and does not impact test performance. The loading dye is removed from the test window as the sample flows through the device.

- Remove the cap from the sample tube and fill the cap with liquid from the tube. Any foam should remain in the tube.
- Dip the head of the Reveal^®^ 3-D for Peanut device into the liquid contained in the cap. Ensure that the cavity of the device is saturated with liquid. Leave the cavity saturated with liquid until liquid is seen running in the device window.
- Place the device on a flat surface and allow the test to develop for 5 min.

### Interpretation of Results

Read the result after 5 min of development. Observations after 6 min may be inaccurate due to overdevelopment of the test.

- *Negative result*.—No line at position T (test). Red lines at positions O (overload) and C (control). Level of peanut is below the detection limit of the test.
- *Positive result*.—A red line of any intensity at position T (test). Red lines at positions O (overload) and C (control). Level of peanut is above the detection limit of the test.
- *High positive result*.—No line at position O (overload) and a faint red line or no line at position T (test). Red line at position C (control). Sample is overloaded with peanut residue.
- *Invalid result*.—No line at position C (control). The test is invalid and should be repeated.

### Further Testing

If desired, samples producing positive Reveal^®^ 3-D for Peanut results may be tested with a validated laboratory assay (e.g., Veratox^®^ for Peanut Allergen, Neogen Corp., or equivalent) to obtain quantitative results.

## Validation Study

Elements of the validation study included a selectivity study, an interference study, a matrix study, a method robustness trial, lot-to-lot consistency and stability testing, and an independent laboratory study to verify select elements of the internal studies. The study was conducted in accordance with the AOAC *ISPAM Guidelines for Validation of Qualitative Binary Chemistry Methods* (4).

### Preparation of Peanut Spiking Solution

NIST SRM 2387 (peanut butter) was used to prepare peanut spiking solution. Twenty-five milligrams of peanut butter were weighed directly into a 50 mL conical bottom polypropylene tube. Twenty-five milliliters of 10 mM phosphate-buffered saline was added and the tube vortexed at high speed for 1 min to create a uniform suspension. Further dilutions of the 1 mg/mL suspension were made in 0.25% bovine gelatin.

For inoculation of environmental surfaces, an alternative procedure was used for preparation of the spiking solution. Fifty milligrams of NIST peanut butter was weighed directly into an extraction bottle. One scoop of extraction additive (Veratox kit) was added followed by 125 mL of 60°C 10 mM PBS. The solution was shaken at 150 rpm in a 60°C water bath for 15 min. The material was allowed to settle for 5 min, then filtered by pouring a minimum of 5 mL through a Whatman #4 filter. Further dilutions were made in 0.25% bovine gelatin.

### Selectivity and Interference by Non-Target Food Materials

- *Methodology.*—The selectivity study was designed to demonstrate that the lateral flow method does not produce positive results with other common food ingredients (cross-reactivity), and also to demonstrate that the method is able to detect peanut in the presence of other food ingredients (lack of interference). Twenty-nine non-target food materials were tested, alone and also with 10 ppm added peanut (Table 1). Food materials were pre-screened for natural contamination with peanut using the Veratox for Peanut Allergen ELISA. Food materials were extracted following instructions for the Veratox^®^ for Peanut Allergen. Five grams of matrix was weighed into an extraction bottle, one level scoop of extraction additive was added, followed by 125 mL of 60°C extraction solution. The bottle was shaken in a 60°C water bath at 150 rpm for 15 min, then allowed to settle for 5 min. A minimum of 5 mL of the extract was then filtered through Whatman #4 filter paper. For each food material, two samples were prepared, one as is, and the other spiked with peanut at 10 ppm. Samples were blind-coded and randomized, then extracted and tested in duplicate following the lateral flow method protocol.
- *Results.*—Results are shown in Table 1. Of the 29 non-target food materials, only one, cashew, produced positive results in the lateral flow test. The cashew sample was tested on Veratox^®^ for Peanut Allergen and was shown to contain 4 ppm peanut. An additional experiment was conducted in which 30 replicates were tested; there were six positive results. A second lot of cashew was obtained and tested. Of 30 test portions, eight tested positive with the Reveal assay. Twelve test portions were also tested with the Veratox assay with results ranging from 0.5 to 14.3 ppm peanut. Although a low level of cross-reactivity of cashew in the Reveal assay cannot be ruled out, these results are most consistent with a low level of peanut contamination of the cashew samples. This is supported by the variability in results between test portions observed with both the Reveal and Veratox assays. Only one food product, walnut, showed interference with the ability of the assay to detect peanut. In walnut samples spiked with 10 ppm peanut, both assays produced negative results. Tests were conducted in which the walnut extract was diluted 1:10, spiked with 10 ppm peanut, and then tested. Duplicate assays were positive, showing that the interference can be avoided with dilution of the test sample. Assay interference would only be expected if the product tested contained >10% walnut by weight. In this case, 1:10 dilution of the extract prior to assay is indicated.

**Table 1.** Results of food commodity selectivity and interference testing for the peanut lateral flow assay.

Commodity	Lateral flow assay results			
	Without added peanut		With 10 ppm peanut	
	Replicate 1	Replicate 2	Replicate 1	Replicate 2
Oat	Negative	Negative	Positive	Positive
Pumpkin	Negative	Negative	Positive	Positive
Brazil nut	Negative	Negative	Positive	Positive
Wheat gluten	Negative	Negative	Positive	Positive
Common wheat	Negative	Negative	Positive	Positive
Barley	Negative	Negative	Positive	Positive
Sunflower	Negative	Negative	Positive	Positive
Soy	Negative	Negative	Positive	Positive
Macadamia nut	Negative	Negative	Positive	Positive
Chick pea	Negative	Negative	Positive	Positive
Green pea	Negative	Negative	Positive	Positive
Coconut	Negative	Negative	Positive	Positive
Pine nut kernel	Negative	Negative	Positive	Positive
Sesame	Negative	Negative	Positive	Positive
Cocoa powder	Negative	Negative	Positive	Positive
Corn	Negative	Negative	Positive	Positive
Buckwheat	Negative	Negative	Positive	Positive
Rye	Negative	Negative	Positive	Positive
Rice	Negative	Negative	Positive	Positive
Chestnut	Negative	Negative	Positive	Positive
Lima bean	Negative	Negative	Positive	Positive
Poppy	Negative	Negative	Positive	Positive
Skim milk powder	Negative	Negative	Positive	Positive
Almond	Negative	Negative	Positive	Positive
Hazelnut	Negative	Negative	Positive	Positive
Pistachio	Negative	Negative	Positive	Positive
Walnut	Negative	Negative	Negative	Negative
Walnut 1:10^a^	Negative	Negative	Positive	Positive
Cashew	Positive	Positive	Positive	Positive
Dark chocolate	Negative	Negative	Positive	Positive

a 1:10 dilution of extract.

### Interference by Clean-in-Place Sanitizer

- *Methodology.*—An experiment was conducted to assess interference from a clean-in-place sanitizer applied to an environmental surface. Working strength quaternary ammonium sanitizer (Quat-Stat^TM^ 5, Betco, Bowling Green, OH; contains dioctyl dimethyl ammonium chloride and alky-dimethyl benzyl ammonium chloride, used at 0.5 oz per gallon of water) was applied to 10 × 10 cm stainless steel surface areas and allowed to dry for approximately 1 h. The surface areas were swabbed, and the swabs extracted according to the standard procedure. Extracts were spiked with peanut at levels ranging from 0 to 20 ppm. Triplicate extracts were prepared at each level and assayed.
- *Results.*—Results are shown in Table 2. All tests conducted on spiked extracts were positive. Line intensity increased with increasing peanut concentration. There was no evidence of assay interference from the CIP rinse.

**Table 2.** Results of CIP rinse interference testing for the peanut lateral flow assay

Spike level, ppm^a^	Lateral flow assay results		
	Replicate 1	Replicate 2	Replicate 3
0	Negative	Negative	Negative
2.5	Positive	Positive	Positive
5	Positive	Positive	Positive
10	Positive	Positive	Positive
20	Positive	Positive	Positive

a Stainless steel was spiked with working strength QuatStat 5 CIP rinse, sampled by swabbing, extracted, spiked with peanut, and tested (see *Materials and Methods*).

### Matrix Study

- *Methodology.*—CIP rinses: Quat-Stat 5 was prepared at 25% working strength in water. TexCide^TM^ (Texwipe, Kernersville, NC; contains 5.9% peroxyacetic acid and 27.3% hydrogen peroxide, 4 oz per gallon of water working strength concentration), was prepared at 1% working strength in water. Results of preliminary experiments demonstrated that these were the highest concentrations of the two CIP rinses that could be tested directly in the lateral flow assay without inhibiting assay activity (data not shown). Even the 1% concentration represents an excess over what would normally be present after multiple water rinses following use of clean-in-place sanitizers in a food manufacturing or food preparation setting. For each material, 33 replicate test portions were prepared at each of five peanut target concentrations (0, 1–2, 3, 5, and 10 ppm) using the peanut spiking solution. In some cases, a 0.5 ppm peanut level was included. At least one level was expected to produce a fractional positive data set (25–75% of test portions positive). Three test portions at each level were reserved for testing with the Veratox^®^ for Peanut Allergen assay to verify the spike levels. The remaining test portions were randomized, blind-coded, and assayed with the lateral flow test.

####  

*Methodology*.—Environmental surface swabs: Diluted peanut spiking solution was used to inoculate plastic and stainless steel 10 x 10 cm surface areas. Approximately 0.25 mL of inoculum was applied. For testing of dry surfaces, the inoculated surfaces were allowed to dry for 16–24 h at 20–24°C. For testing of wet surfaces, the inoculated surfaces were allowed to dry for 30–60 min, but were still visibly wet when sampled. For each surface and condition (wet or dry), the following replicate test portions were prepared: 30 at a peanut level of 1 μg/100 cm^2^, 30 at a level of 2 μg/100 cm^2^, five at a level of 5 μg/100 cm^2^, and five controls without peanut. Again, at least one level was expected to produce fractional positive results. The surface areas were swabbed and extracted. The extracts were randomized, blind-coded, and tested with the lateral flow assay.

POD with a 95% confidence interval was calculated per matrix and spiking level using an online calculator (3).

(b)*Results.*—CIP rinses: Results for CIP rinses are shown in Table 3. Water and the peroxyacetic acid/hydrogen peroxide sanitizer (1% working concentration) produced nearly identical results; POD was 0.6 at 0.4–0.5 ppm peanut, 0.9 at 0.8–1.0 ppm peanut, and 1 at 2.0–2.8 ppm peanut. With the quaternary ammonium sanitizer (25% working concentration), the test was slightly less sensitive; POD was 0.4 at 0.7 ppm peanut, 0.97 at 2.2 ppm peanut, and 1 at 4.2 ppm peanut.

**Table 3.** Results of CIP rinse testing for the peanut lateral flow assay

Sample	Target spike level, ppm	Actual spike level, ppm^a^	No. test portions	No. positive	POD, 95% CI^b^
Water	0	0	30	0	0 (0–0.11)
	0.5	0.5	30	18	0.60 (0.42–0.75)
	1.5	1.0	30	27	0.90 (0.74–0.97)
	3	2.8	30	30	1 (0.89–1)
	5	4.3	30	30	1 (0.89–1)
	10	11.6	30	30	1 (0.89–1)
Peroxyacetic acid/hydrogen peroxide (1% working strength)	0	0	30	0	0 (0–0.11)
	0.5	0.4	30	17	0.57 (0.39–0.73)
	1.5	0.8	30	27	0.90 (0.74–0.97)
	3	2.2	30	30	1 (0.89–1)
	5	6.7	30	30	1 (0.89–1)
	10	25.6^c^	30	30	1 (0.89–1)
Quaternary ammonium (25% working strength)	0	0	30	0	0 (0–0.11)
	1	0.7	30	12	0.40 (0.25–0.58)
	3	2.2	30	29	0.97 (0.83–1)
	5	4.2	30	30	1 (0.89–1)
	10	10.1	30	30	1 (0.89–1)
Quaternary ammonium^d^ (25% working strength)	0	0	30	0	0 (0–0.11)
	1–2	0.9	30	8	0.27 (0.14–0.44)
	3	3.1	30	23	0.77 (0.59–0.88)
	5	4.5	30	30	1 (0.89–1)
	10	10.9	30	30	1 (0.89–1)

a Determined using the Veratox^®^ for Peanut Allergen quantitative assay.

b Probability of detection with 95% confidence interval.

c Spike was known to be incorrect.

d Trial performed by independent laboratory.

*Results*.—Environmental surface swabs: Results for environmental surface swabs are shown in Table 4. Swabs taken from wet and dry stainless steel surfaces produced similar results with POD of 0.8–0.9 at approximately 2 ppm peanut and POD of 1 at 3–4 ppm peanut, respectively. With swabs from plastic, POD was >0.9 at 2 ppm peanut and 1 at 3 ppm peanut for both the wet and dry surfaces.

**Table 4.** Results of environmental surface testing for the peanut lateral flow assay

Sample	Target spike level, µg/100 cm^2^	Actual spike level, µg/100 cm^2^^a^	No. test portions	No. positive results	POD, 95% CI^b^
Stainless steel-wet	0	0	5	0	0 (0–0.43)
	1	0.95	30	7	0.23 (0.12–0.41)
	2	2.0	30	23	0.77 (0.59–0.88)
	3	3.0	30	30	1 (0.89–1)
	5	4.8	5	5	1 (0.57–1)
Stainless steel-wet^c^	0	0	5	0	0 (0–0.43)
	1	1.0	30	19	0.63 (0.46, 0.78)
	2	2.0	30	30	1 (0.89, 1)
	5	5.0	5	5	1 (0.57, 1)
Stainless steel-dry	0	0	5	0	0 (0, 0.43)
	1	1.4	30	20	0.67 (0.49, 0.81)
	2	1.9	30	27	0.90 (0.74, 0.97)
	3	3.0	30	29	0.97 (0.83, 1)
	5	4.5	5	5	1 (0.57, 1)
	0	0	5	0	0 (0, 0.43)
	1	1.0	30	12	0.40 (0.25, 0.58)
Stainless steel-dry^c^	2	2.0	30	30	1 (0.89, 1)
	5	5.0	5	5	1 (0.57–1)
Plastic-wet	0	0	5	0	0 (0–0.43)
	1	1.2	30	10	0.33 (0.19–0.51)
	2	2.0	30	29	0.97 (0.83–1)
	3	3.0	30	30	1 (0.89–1)
	5	5.0	5	5	1 (0.57–1)
	0	0	5	0	0 (0–0.43)
	1	1.2	30	8	0.27 (0.14–0.44)
Plastic-dry	2	2.0	30	28	0.93 (0.79–0.98)
	3	3.0	30	30	1 (0.89–1)
	5	5.0	5	5	1 (0.57–1)

a Determined using the Veratox^®^ for Peanut Allergen quantitative assay.

b Probability of detection with 95% confidence interval.

c Trial performed by independent laboratory.

### Robustness Testing

- *Methodology.*—The ability of the lateral flow assay to withstand modest variation in operating parameters was assessed in a robustness experiment. Three parameters were chosen for investigation: *1*) extraction buffer temperature (4–8°C, 30–35°C, and the standard condition of 22–26°C), *2*) extraction shaking time (45 s, 75 s, and the standard condition of 60 s), and *3*) lateral flow device development time (4.5 min, 5.5 min, and the standard condition of 5 min). Variations were tested in combination in a nine-condition matrix experiment (Table 5). Water was used as the sample material. Ten replicate test portions spiked with 1 ppm peanut served as the positive sample; this spike level was expected to produce a fractional positive data set. Ten replicate test portions of water served as the negative sample. Test portions were randomized, blind-coded, and tested in the lateral flow assay. POD analysis was conducted comparing results at each condition containing variations to the nominal condition.
- *Results.*—Results are shown in Table 5. The nominal condition produced 90% positive results. The eight conditions with assay parameter variations produced between 50 and 90% positive results. Even in the case of condition eight, 50 versus 90% positive results, the difference was not significant by POD analysis at *P* < 0.05 [dPOD = -0.40 (-0.68, 0.00)].

**Table 5.** Results of robustness testing for the peanut lateral flow assay

				% Positive results^a^	
Condition	Extraction buffer temperature, ^o^C	Extraction shake time, s	Development time, min	Negative sample^b^	Positive sample^c^
1	4–8	45	4	0	90
2	4–8	45	6	0	90
3	4–8	75	4	0	80
4	4–8	75	6	0	70
5	30–35	45	4	0	80
6	30–35	45	6	0	80
7	30–35	75	4	0	70
8	30–35	75	6	0	50
9^d^	22–26	60	5	0	90

a Ten replicates were tested.

b Negative sample – water.

c Positive sample – water spiked with 1 ppm peanut using NIST peanut butter standard.

d Nominal conditions for the assay.

### Product Consistency and Stability Studies

- *Methodology*.—Three manufactured lots of Reveal^®^ 3-D for Peanut test kits were tested at time points between 0 and 23 months post-manufacture.
- *Results.* –There was no indication of a significant difference in performance between lots, or of a decreasing positivity rate over the course of the stability study. Product expiration dating of up to 23 months is supported.

### Independent Laboratory Study

- *Methodology.*—Trials were conducted with peanut-spiked quaternary ammonium-based CIP rinse (Bacdown^®^ Detergent Disinfectant, Decon Labs, Inc., King of Prussia, PA; contains n-alkyl dimethyl benzyl ammonium chloride and n-alkyl dimethyl ethylbenzyl ammonium chloride) at 25% working concentration, and with wet and dry stainless steel surfaces inoculated with peanut. Procedures followed those described above for the internal validation trials, including the use of the NIST peanut butter standard to prepare the spiking materials.
- *Results*.—Results are shown in Tables 3 and 4. For the CIP rinse (quaternary ammonium sanitizer), results were similar to those of the internal trial. POD was 0.8 at 3.1 ppm peanut and 1 at 4.5 ppm peanut. For swabs from wet and dry stainless steel surfaces, results were again consistent with those of the internal trials. POD was 0.4–0.6 at 1 ppm peanut and 1 at 2 ppm peanut.

## Discussion

Results of the validation study reported here demonstrate that the Reveal^®^ 3-D for Peanut lateral flow test is a sensitive and specific method for determination of peanut residue in CIP rinses and in swabs from environmental surfaces. For CIP rinses, including water, peroxyacetic acid/hydrogen peroxide (1% working strength), and quaternary ammonium (25% working strength) sanitizers, considering all trials, POD was 1 in the range of 2–4 ppm peanut. For surface swabs from stainless steel and plastic, POD was 1 at 3–4 ppm. Results of the independent laboratory study with CIP rinse and wet and dry stainless steel surfaces were consistent with those of the internal trials.

Specificity testing with 29 common food commodities showed the assay to be very specific, with no clear evidence of cross-reactivity. Walnut was shown to interfere with the assay’s ability to detect peanut at 10 ppm, although the interference can be avoided with 1:10 dilution of the sample prior to assay. As long as the CIP rinse or environmental surface swab contains <10% walnut, test kit users can be confident that there will be no interference. Residual quaternary ammonium sanitizer did not interfere with the ability of the assay to detect low levels of peanut in swabs from a stainless steel surface. Results of robustness testing showed that the assay is able to withstand modest variation in multiple operating parameters simultaneously. Results of stability and consistency testing trials support expiration dating for the Reveal^®^ 3-D for Peanut test kit of at least 23 months.

## Conclusions

Results of the validation study demonstrate that the Reveal^®^ 3-D for Peanut test is an accurate, sensitive, and specific method for qualitative detection of peanut in CIP rinses and environmental surface samples. Further, the test is simple to perform, requiring no specialized equipment and providing results in less than 10 min, including sample extraction. The test provides a powerful tool to food manufacturing and food preparation personnel for management of peanut allergen risk. It is recommended that the Reveal^®^ 3-D for Peanut test kit be granted AOAC *Performance Tested Method* status.

## Supplementary Material

qsz041_Supplementary_Data

## Certification Information

The method was independently tested, evaluated, and certified by the AOAC Research Institute as a Performance Tested Method^SM^. See https://www.aoac.org/scientific-solutions/research-institute-ptm/ for information on certification.
