# Supplementary material for: Validation of the Reveal® 3-D for Peanut Lateral Flow Test: AOAC Performance Tested MethodSM 111901
Source: J AOAC Int. 2020 Jul 23;103(4):1112–8. doi: 10.1093/jaoacint/qsz041 (PMC8370410; doi:10.1093/jaoacint/qsz041)
Supplement: qsz041_Supplementary_Data [file qsz041_supplementary_data.pdf]

AOAC INTERNATIONAL

MANUSCRIPT SUBMISSION APPROVAL AND COPYRIGHT TRANSFER

Type or print the following information: date 11/26/19

TITLE Validation of the Reveal 3D for Peanut Lateral Flow Test

AUTHOR(S) Quynh-Nhi Le, Alexis Vance, Nawal Bakir Dave Almy, Emily Slenk, Brooke Roman, Robert Donofrio

Transfer of copyright. In the event that this article is accepted for publication in the Journal of AOAC INTERNATIONAL, the Author(s) hereby transfers to AOAC INTERNATIONAL all copyrights and certifies that he/she is authorized to do so. Return rights. In return, AOAC INTERNATIONAL hereby grants to the above-named Author and his/her employer royalty-free license to retain all patent rights, and to use the article provided that author shall, as a condition of such license, place the appropriate copyright notice of AOAC INTERNATIONAL on each such use and indicate that author has a license from AOAC INTERNATIONAL to use the article. Note: **Redistribution for commercial purposes or for social networking is strictly prohibited. Violation of this copyright agreement may result in prosecution to the fullest extent of the law.**

The copyright to this article is transferred to AOAC INTERNATIONAL effective if and when the article is accepted for publication. The copyright transfer covers the exclusive right to reproduce and distribute the article, including reprints, translations, photographic reproductions, microform, electronic form (offline, online) or any other reproductions of similar nature. Author certifies that the manuscript has been read and approved for submission.

NOTE: ALL AUTHORS MUST SIGN EITHER PART A OR PART B. IF ALL AUTHORS SIGNATURES DO NOT APPEAR ON THE FORM, THE SENIOR AUTHOR MUST SIGNIFY THAT HE/SHE HAS THE AUTHORITY TO SIGN FOR THOSE AUTHORS WHOSE SIGNATURES DO NOT APPEAR ON THE FORM.

NO MANUSCRIPT WILL BE PUBLISHED IN THE *JOURNAL OF AOAC INTERNATIONAL* UNLESS THIS SIGNED FORM IS RETURNED

PART A

Transfer of copyright to above-named article by academic, industry, and other nongovernmental Authors:

Author signature and date [signature] 11-26-19

Type or print name Quynh-Nhi Le

Author signature and date

Type or print name Alexis Vance

Author signature and date [signature] 11-27-19

Type or print name ~~Nawal Bakir~~ QL 12-10-19

Author signature and date [signature] 12-10-19

Type or print name Dave Almy

Author signature and date [signature] 12-9-19

Type or print name Emily Slenk

Author signature and date [signature] 12-6-19

Type or print name Brooke Roman

AOAC INTERNATIONAL

Author signature and date Author signature and date Robert [illegible]

Type or print name Type or print name Robert Donofrio

I certify that I have the authority to sign for those authors whose name(s) do not appear on this form:

Senior Author signature and date Robert [illegible]

Type or print name Robert Donofrio

Part B Certification that the above-named article was prepared by a government employee as part of his/her official duties or under a government grant specifically prohibiting copyright and therefore cannot be copyrighted.

Author signature and date Author signature and date ______________________

Type or print name Type or print name ______________________

Author signature and date Author signature and date ______________________

Type or print name Type or print name ______________________

Author signature and date Author signature and date ______________________

Type or print name Type or print name ______________________

I certify that I have the authority to sign for those authors whose name(s) do not appear on this form:

Senior Author signature and date ______________________

Type or print name ______________________

AOAC INTERNATIONAL

Statement of Authorship

Document TITLE:____

The undersigned authors hereby confirm that:

- *This manuscript has been submitted solely to this journal and is not published, in press, or submitted elsewhere.*
- *This manuscript is an original work without fabrication or plagiarism.*
- *All authors have read and approved the manuscript and are prepared to take public responsibility for the work*
- *This manuscript identifies all co-authors who have substantially contributed to the concept, data collection and analysis or preparation of the manuscripts and therefore who may have intellectual property claims to the content. The authors attest that those individuals or organizations mentioned in the Acknowledgements are aware that their names appear in the manuscript.*
- *All authors have declared all sources of funding for the work reported in their manuscript and reported all potential conflict of interest.*

Senior Author signature and date Robert D[illegible]

Type or print name Robert Donofrio
